# Factors influencing the implementation of integrated screening for HIV, syphilis, and hepatitis B for pregnant women in Nepal: A qualitative study

**DOI:** 10.1371/journal.pgph.0003006

**Published:** 2024-10-11

**Authors:** Lucie Sabin, Hassan Haghparast-Bidgoli, Bibhu Thapaliya, Obindra Chand, Sanju Bhattarai, Abriti Arjyal, Naomi Saville

**Affiliations:** 1 Institute for Global Health, University College London, London, United Kingdom; 2 Swisscontact, Kathmandu, Nepal; 3 School of Health and Social Care, University of Essex, Colchester, United Kingdom; 4 Department of Public Health and Nursing, Norwegian University of Science and Technology, Trondheim, Norway; 5 HERD International, Kathmandu, Nepal; University of Toronto, CANADA

## Abstract

In Nepal, national guidelines recommend free HIV and syphilis screening for pregnant women at their first antenatal visit, using an opt-out approach. However, screening uptake is low and the guidelines do not include hepatitis B screening. It is essential to understand the factors influencing the implementation of integrated screening for HIV, syphilis, and hepatitis B, as recommended by WHO, to improve uptake and prevent vertical transmission. This study explored the knowledge, attitudes, and perceptions of pregnant women, their families, healthcare providers and policymakers on integrated prenatal screening. We conducted 12 in-depth interviews with pregnant women, 10 with their husbands and 4 with mothers-in-law in Kapilvastu and Kathmandu. In addition, we interviewed 7 health workers and 4 decision-makers. These interviews were sufficient to reach saturation. Data were analysed using a thematic content analysis. A combination of the social-ecological model and the WHO building blocks provided a theoretical framework for interpreting data. The analysis showed that antenatal screening in Nepal involved many stakeholders and was influenced by various factors. Implementation issues were found in the building blocks service delivery, health workforce and medical products. Husbands and in-laws play an important role in the acceptance of screening by pregnant women, especially in rural areas. High levels of stigma and discrimination against people with sexually transmitted diseases were reported, and knowledge of hepatitis B and syphilis was low. Access and uptake of screening could be improved through rapid testing, by strengthening the health system and by integrating hepatitis B screening through an opt-out approach like that for HIV and syphilis. Effective community involvement through awareness campaigns and investment in lower-level health facilities is essential to improve screening rates. This study provides information for decision-makers about challenges in implementing integrated screening to guide the design of targeted interventions to reduce vertical transmission.

## Introduction

Human immunodeficiency virus (HIV), syphilis and hepatitis B contribute significantly to the global burden of disease. HIV, syphilis and hepatitis B can be transmitted from mothers living with the disease to their children during pregnancy and childbirth also known as vertical transmission. Hepatitis B can also be transmitted horizontally during childhood. In 2020 in Nepal, HIV prevalence was estimated at 0.12% among adults aged 15–49 years [1]. Hepatitis B prevalence was estimated at 5% among pregnant women in 2019 in Nepal [2]. Syphilis prevalence estimates vary, with an estimated 1.5% among a group of 3,570 women screened [3] and 0.2% among 1,362 women screened [4].

Unknowingly women living with these sexually transmitted infections (STIs) risk transmitting diseases to their sexual partners or children through vertical transmission. They may also miss timely treatment, leading to long-term health complications and increased healthcare costs. Screening during antenatal care (ANC) is key to eliminating vertical transmission. Early detection and treatment of these diseases in pregnant women can significantly reduce the risk of vertical transmission [5–7]. Since 2010, vertical transmission interventions have prevented around 7,400 new HIV diseases in children in the Asia-Pacific region [8]. To promote the triple elimination of vertical transmission of HIV, syphilis and hepatitis B, the World Health Organisation (WHO) has developed an Asia-Pacific regional framework [9]. This framework aims to eliminate these diseases in newborns and infants in Asia by 2030, via a focus on maternal, newborn and childcare. The framework encourages an integrated approach to triple elimination, recognising the interconnectedness of the three diseases and the potential for resource optimisation. Integrated antenatal screening for HIV, syphilis and hepatitis B allows pregnant women to be tested for these three diseases as well as performing the entire ANC panel with a single blood sample.

In Nepal, the National HIV Testing and Treatment Guidelines provide free HIV screening for all pregnant women during ANC visits following an opt-out approach [10]. In addition, according to the Nepal Safe Motherhood and Newborn Health Roadmap 2030 [11], systematic screening for HIV and syphilis should be offered to all pregnant women during the first ANC visit. Despite the efforts made, the country is still far from achieving the triple elimination targets set by the WHO [12]. In 2020, only 65% of pregnant women had a known HIV status, and only 40% of pregnant women living with HIV were receiving antiretroviral treatment to reduce the risk of vertical transmission, compared with the target of 95%. Coverage of syphilis screening among women receiving antenatal care (ANC) in 2018 was very far below the 95% target at 0.03% [13]. However, the few ANC attendees who tested positive for syphilis received appropriate treatment. No data are available on the coverage of hepatitis B screening among people who received antenatal care, and no national guidelines cover hepatitis B screening.

Many stakeholders are involved in the screening of HIV, syphilis and hepatitis B amongst pregnant women including healthcare workers on the supply side and pregnant women’s family members on the demand side. However, few studies in Nepal investigated the demand side [14, 15] and supply side [16] factors affecting antenatal screening of HIV. Thapa et al. (2019) [15] examined the association between women’s empowerment and HIV testing among Nepali women using data from the 2012 Nepal Demographic and Health Survey. They found that age, education, and wealth were factors associated with HIV testing among women. Pokharel, Abbas, et Ghimire (2011) [14] studied the implementation of the prevention of vertical transmission programme and found that the acceptability of the test after counselling was satisfactory. They stressed the need for greater male involvement. Only one study examined supply-side barriers. Using the 2015 Nepal Health Facility Survey (NHFS), Acharya et al. (2020) [16] showed that the readiness of facilities for HIV counselling and testing services was higher than for other STI services. They found persistent gaps in staffing, guidelines, drugs, and commodities in both services.

By interviewing pregnant women, their family members, health workers and decision-makers, this study aimed to explore factors influencing the implementation and the acceptance of integrated antenatal screening of HIV, syphilis, and hepatitis B in Nepal. This study can provide useful information for decision-makers regarding the challenges and facilitators affecting integrated screening. It also can guide the design of interventions to improve antenatal screening rates in the country by addressing potential barriers. Additionally, it can contribute to the global effort to improve maternal and child health by promoting integrated antenatal screening for HIV, syphilis, and hepatitis B.

## Methods

### Setting

Nepal’s health budget has risen from NRs 23.81 billion in 2011 to NRs 122.79 billion in 2022, and the share of health expenditure in consolidated public spending has reached 7.5% of GDP. In 2021, external healthcare expenditure and out-of-pocket expenditure remained important, representing respectively 10% and 54% of current healthcare expenditure.

Many health workers from different health facilities are involved in the provision of screening for STIs for pregnant women in Nepal. Female Community Health Volunteers (FCHVs) play an important role in antenatal screening in rural communities. They link communities and the health system, providing basic health services and raising awareness on health issues. Health posts are the most available type of institution for primary healthcare in the communities across Nepal. Women go there for registration, ANC visits, screening, and immunisation as part of their pregnancy. Public hospitals have a larger capacity than health posts and have more screening facilities. They are owned and operated by the government, while private hospitals are owned and operated by private entities. Other essential actors in antenatal screening in Nepal are non-governmental organisations (NGOs). Some are involved in screening at-risk individuals, treating, and monitoring positive cases.

For the purposes of our study, it is important to understand the differences between the Kathmandu Valley and Kapilvastu district where our study took place.

The Kathmandu Valley, with Kathmandu the federal capital city of Nepal, is the most developed urban area of Nepal with more than 1.5 million population [17]. Located in Bagmati province in the hills, the agglomeration attracts migrants from all over the country. The Kathmandu Valley houses many of the health facilities in the country with 30 public hospitals including the Paropakar Maternity and Women’s Hospital (PMWH) (the national referral centre for obstetrics), numerous other tertiary referral hospitals and 82 health posts [18].

Kapilvastu is a *Terai* (plains) district, which has a 55-bed government district hospital and 12 health posts. Bordering India, its population comprises largely Madhesi ethnic groups including a high proportion of Muslims and Dalits, who are often marginalised and face discrimination [19]. Male foreign labour migration to India, the Middle East and East Asian countries (especially Malaysia) [20] is widespread in this district which increases vulnerability to STIs. Most of the population are Awadhi-speaking rural farmers [21]. The Terai area of Nepal has a low Human Development Index (HDI) score largely driven by poor education indicators [22] and the highest levels of gender disparity in Nepal [19].

### Participants recruitment

Characteristics of participants are presented in Table 1. Participants were recruited between 10 January 2023 and 7 April 2023. On the demand side, we conducted a total of 14 semi-structured in-depth interviews in Kapilvastu and 12 in Kathmandu with pregnant women and their household members (i.e. husbands and mothers-in-law). We interviewed 12 currently pregnant women (6 in Kapilvastu and 6 in Kathmandu) and since joint households are widespread in Kapilvastu, we also interviewed 4 mothers-in-law (Table 2). To explore male spouses’ perspectives on maternal health and antenatal screening for STIs we interviewed 10 husbands (4 in Kapilvastu and 6 in Kathmandu), among them, five were husbands of pregnant women who had been interviewed.

**Table 1:** Participants and required number of Semi-Structured Interviews (SSI).

	Activity	Criteria of selection		
**Demand side**			**Kathmandu**	**Kapilvastu**
Pregnant women	12 SSIs	Woman from disadvantaged caste.	3	3
		Woman from high caste.	3	3
Husbands	10 SSIs	Husband from disadvantaged caste.	3	2
		Husband from high caste.	3	2
Mothers-in-law	4 SSIs	Mother-in-law from disadvantaged caste.	-	2
		Mother-in-law from high caste.	-	2
**Total**			**12**	**14**
**Supply side**				
Health workers	7 SSIs	Health worker from public hospital	1	1
		Health worker from a stand-alone HIV testing site.	1	1
		Health worker from a health post.	1	1
		Health worker from a private hospital.	1	-
Decision-makers	4 SSIs	Rep’ from the Ministry of Health.	1	-
		Rep’ from the health division of the Municipality.	-	1
		Rep’ from the Nepal Health Sector Support Program.	1	-
		A person implementing programs for the Global Fund.	1	-
**Total**			**7**	**4**

Notes: Disadvantaged castes included Dalit, Tharu and Muslim. High castes included Baniya, Brahmin, Chhetri.

**Table 2:** Pregnant women, husbands and mother-in-law’s characteristics.

Household characteristics		Pregnant woman characteristics					Husband characteristics				Mother-in-law characteristics			
**Type of household**	**Husband abroad?**	**N°**	**Age**	**Education**	**Occupation**	**Gestational age**	**N°**	**Age**	**Education**	**Occupation**	**N°**	**Age**	**Education**	**Occupation**
Joint	Yes	**1**	21	7	Housewife	9	**1**	23	9	Seller	**1**	45	0	Housewife
Joint	No	**2**	22	9	Housewife	2	**-**	-	-	-	**-**	-	-	-
Joint	No	**3**	27	12	Housewife	2	**-**	-	-	-	**2**	59	0	Housewife
Joint	Yes	**4**	38	10	Housewife	1	**2**	28	10	Unemployed	**-**	-	-	-
Joint	No	**5**	23	7	Housewife	5	**-**	-	-	-	**3**	52	0	Housewife
Nuclear	No	**6**	24	0	Housewife	9	**3**	42	0	Daily wage worker	**-**	-	-	-
Joint	Yes	**-**	-	-	-	8	**4**	45	8	Real Estate Broker	**-**	-	-	-
Joint	Yes	**-**	-	-	-	9	**-**	-	-	-	**4**	55	0	Housewife
Nuclear	No	**7**	33	2	Housewife	7	**5**	39	SLC	Insurance Broker	**-**	-	-	-
Nuclear	No	**8**	24	12	Lab Assistant	9	**-**	-	-	-	**-**	-	-	-
Nuclear	No	**9**	28	SLC	Beautician	6	**-**	-	-	-	**-**	-	-	-
Joint	No	**10**	26	SLC	Housewife	8	**-**	-	-	-	**-**	-	-	-
Nuclear	No	**11**	33	Master	Teacher	9	**-**	-	-	-	**-**	-	-	-
Nuclear	No	**12**	26	5	Housewife	6	**6**	38	8	Seller	**-**	-	-	-
Joint	No	-	-	-	-	6	**7**	21	12	Student	**-**	-	-	-
Nuclear	No	-	-	-	-	9	**8**	24	10	Daily wage worker	**-**	-	-	-
Joint	No	-	-	-	-	3	**9**	30	SLC	Pathao driver	**-**	-	-	-
Nuclear	No	-	-	-	-	7	**10**	28	8	Construction worker	**-**	-	-	-

Notes: Joint household is a family structure in which several generations live together under the same roof. In Nepal, newly-married women often move in with their in-laws family. In contrast to joint households, nuclear households consist solely of parents and their children.

In Kapilvastu, we contacted pregnant women through the Female Community Health Volunteers (FCHVs) purposively approached in one semi-urban municipality (Kapilvastu) and two rural municipalities (Shuddhodhan and Maya Devi). Pregnant women were recruited based on the selection criteria presented in Table 1 and defined before recruitment, ensuring they met the inclusion criteria. A trained and experienced qualitative researcher (BT) worked with five females and a male local interviewer to locate and interview pregnant women and their family members. They interviewed people of the same gender respectively. We trained local interviewers based on available literature on how to conduct interviews on sensitive topics [23, 24]. We conducted nine interviews in the local language Awadhi and five in Nepali. A previous study in a similar context overcame the reluctance of mothers-in-law to let their daughters-in-law be interviewed in private by implementing a parallel interview strategy [25]. We applied a similar strategy by conducting concurrent but separate interviews in two different locations of the household with pregnant women and their mothers-in-law. We interviewed all the husbands alone around the household.

In Kathmandu, we identified and approached participants during their visits to the ANC clinics of the PMWH. A gynaecologist from the hospital introduced us and the study to the participants in the ANC unit. Pregnant women and husbands were interviewed in a café nearby where we could ensure privacy respectively by BT and a male qualitative researcher (OC) trained for this study. Participants gave their written consent to participate. They were not reimbursed for their time. Pregnant women and husbands were relatively reluctant to participate in the study as we met them in the hospital setting, they were often rushing to go home after their check-ups. 2 pregnant women and 4 husbands refused consent. We audio recorded all interviews which lasted 30 minutes to 2 hours.

On the supply side, we interviewed health workers involved in antenatal screening and local and national health system decision-makers. Health workers and decision-makers were recruited based on their roles and ability to answer specific questions regarding antenatal screening for STIs. We conducted 4 interviews in Kapilvastu and 7 in Kathmandu. Participant characteristics are presented in Tables 3 and 4.

**Table 3:** Health workers’ characteristics.

№	Organisation	Municipality	Occupation	Years of experience	Gender
1	Health post	Kapilvastu	Auxiliary Nurse Midwife	6	Female
2	NGO	Kapilvastu	Counsellor at ART Centre	5	Female
3	Public hospital	Kapilvastu	Nursing Officer	7	Female
4	Health post	Kathmandu	Auxiliary Nurse Midwife	23	Female
5	Public hospital	Kathmandu	Doctor	7	Male
6	Public hospital	Kathmandu	Auxiliary Nurse Midwife	38	Female
7	Private hospital	Kathmandu	Registrar in Obstetrics and Gynaecology	3	Female

**Table 4:** Decision makers’ characteristics.

№	Organisation	Occupation	Years in current job
1	Kapilvastu Municipality Office, Health Branch	Health Coordinator	7
2	Ministry of Health and Population, Family Welfare Division	Head of the Maternal and Newborn Health Section	3 months
3	Recovering Nepal	Technical Advisor	15
4	Nepal Health Sector Support Programme	Former technical advisor	10

We interviewed consenting mothers-in-law and husbands of a sampled pregnant woman. In Kapilvastu, the strategy of interviewing pregnant women, husbands and mothers-in-law from the same family was prioritized to allow triangulation resulting in a household with all three household members interviewed, three with mother-in-law and pregnant women only interviewed and three with husband and pregnant women interviewed. Since not all women’s husbands and mothers-in-law were available at the time of interviews, husbands and mothers-in-law from different families were contacted until the sample size was met. The sample size was determined based on the concept of saturation, according to which data collection continues until no new themes emerge [26]. This strategy of interviewing people from the same household was not appropriate in the Kathmandu urban context as pregnant women were reluctant to talk knowing that their partner would also be interviewed. Although pregnant women were usually accompanied by their husbands, the latter were in a hurry to get back to work. We were unable to interview mothers-in-law because they did not accompany pregnant women on ANC visits. Saturation was reached by the end of the data collection and new analytical insights from additional interviews became rare.

Pregnant women and their families were interviewed about their experience of ANC visits, blood tests, knowledge of and behaviours toward STIs and antenatal screening for STIs during pregnancy. Our topic guides evolved after the pilot phase and over time, to explore emergent themes over the course of data collection. The pilot phase consisted of a few interviews with pregnant women, their husbands and mothers-in-law in Kathmandu and Kapilvastu to identify any misunderstandings or difficulties with the questions. These interviews were not included in the analyses. Vignettes, which allow for less personal exploration of sensitive topics that participants might otherwise find difficult to discuss [27–29], were used to interview pregnant women and mothers-in-law. In the vignette, participants were presented with a hypothetical situation in which they were discussing ANC visits and blood tests with a friend. In this hypothetical situation, the friend was also asking for advice about a genital discharge and the possibility of an STI. They aimed to reveal people’s beliefs and potential behaviours without being too intrusive. The vignette was designed according to the local context and on the advice of local researchers and is presented in S1 File.

### Data analysis

**Fig 1:**
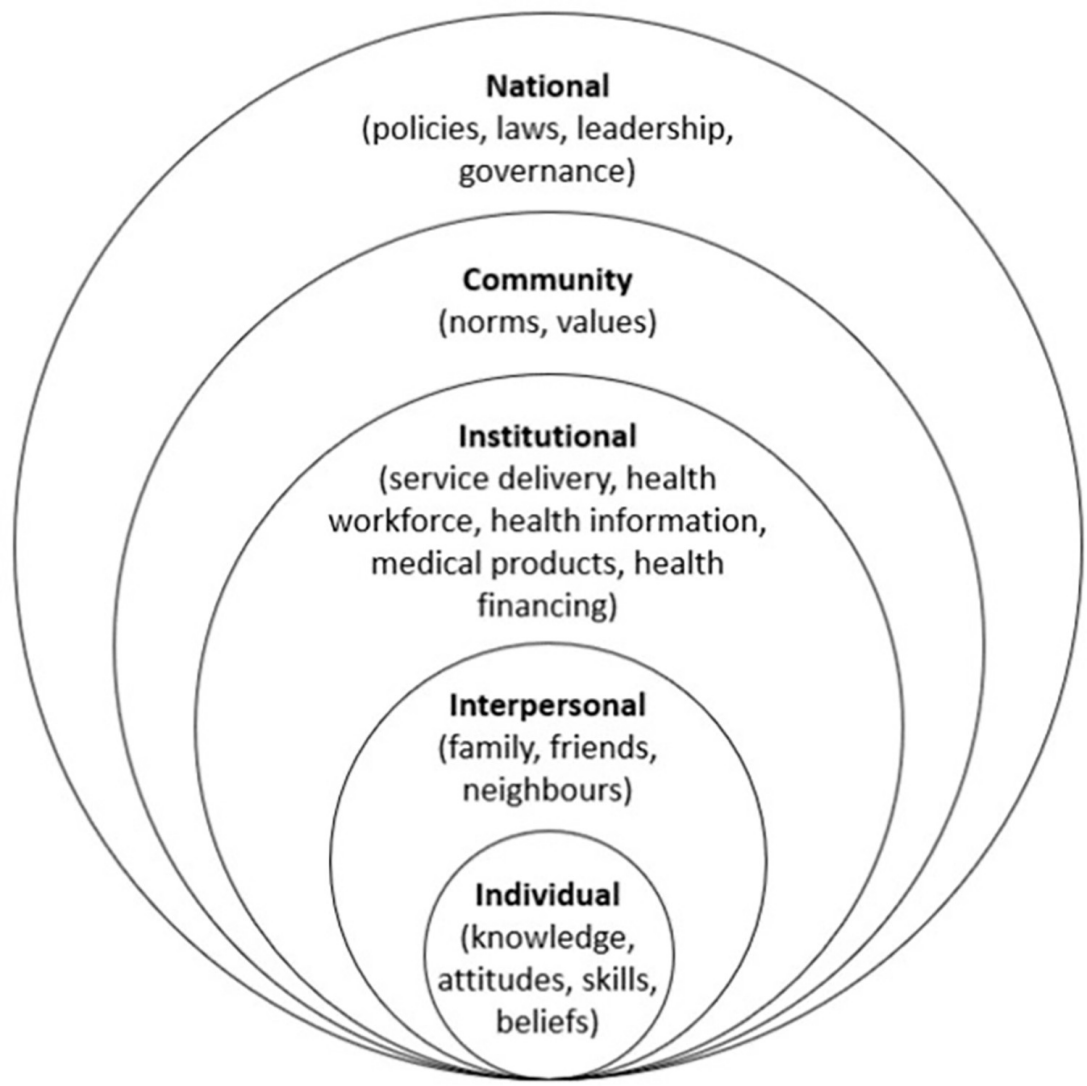
Combination of the social-ecological model and the WHO building blocks.

Data were analysed by LS and BT using NVivo to undertake thematic content analysis [30]. A combination of the social-ecological model and the WHO building blocks provided a theoretical framework for interpreting the data (Fig 1). The social-ecological model proposes a holistic approach and considers the relationship between individuals and their environment to understand health-related behaviours [31]. The model acknowledges that an individual’s behaviour is shaped through complex and interrelated level factors. In this study and according to McLeroy et al. (1988) [31], five levels that influence behaviour will be considered. The first level of influence is the individual level. This level refers to the personal characteristics of an individual that influence their behaviour such as age, gender, ethnicity, knowledge, attitudes, skills, and beliefs. The second is the interpersonal level, which describes the relationships between individuals and the social networks they belong to, such as family, friends, and neighbours. The third is the institutional level which includes formal and informal structures of health in this particular study. The fourth is community level, which refers to established norms and values, standards, and social networks. The higher level of influence is the national level, which describes how the laws, regulations, and policies affect behaviour by creating or limiting opportunities and resources, shaping social norms, and changing the physical and social environment. The WHO building blocks approach [32] was also used to analyse the data from the supply-side perspective and was integrated into the social-ecological framework. This approach provides a framework to improve the performances of health systems by considering six constituent elements: service delivery, health workforce, health information, medical products, health financing, and leadership and governance.

After a step of familiarisation with the data, we read transcripts and carried out a line-by-line analysis by applying the different categories of the social-ecological framework and the WHO building blocks in NVivo. To limit cultural bias and ensure rigour, we double-coded some of the transcripts before finalising the main thematic framework and coding transcripts in NVivo. To develop a deeper analysis of the data, we also conducted a framework analysis [33]. The codes identified in the thematic analysis were applied to the whole data in a systematic way. The themes were compared across the transcripts and specifically the different groups, to establish the range and similarities of the participants’ perceptions, experiences and views. We tabulated quotes from the transcripts within and between transcripts to triangulate the data. We reported results in accordance with qualitative research guidelines [34, 35] and the COREQ checklist [34].

This study has been conducted under an extension of the UCL Global Engagement funding for the Comprehensive Anaemia Programme of Preventive Therapies (CAPPT) project. We received ethical approval for this study from the Nepal Health Research Council (523/2022 P) and University College London Ethics Committee (14301/001).

## Results

**Fig 2:**
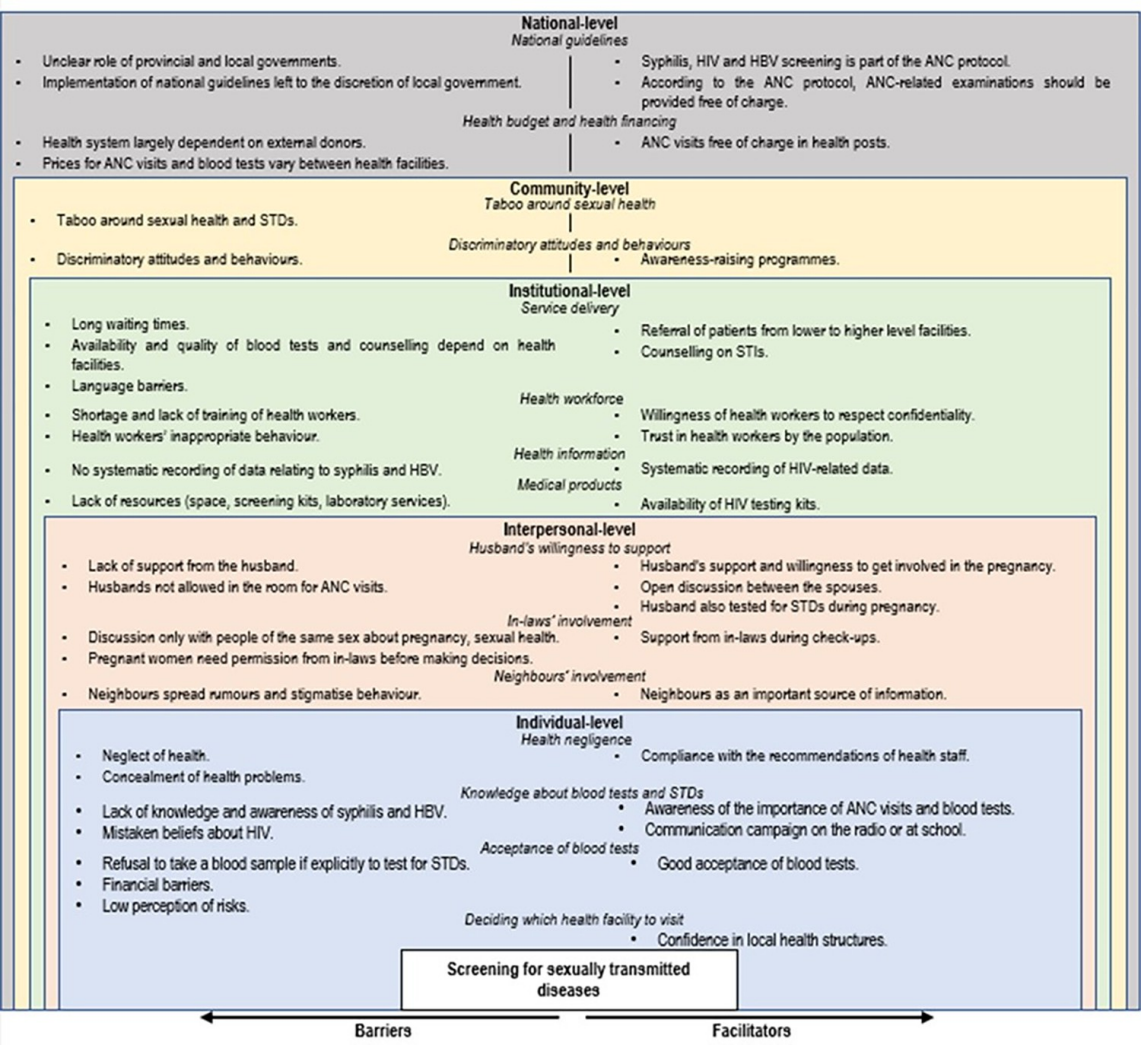
Factors influencing access to and provision of antenatal screening for HIV, syphilis and hepatitis B in Nepal presented according to the social-ecological model and the WHO building blocks.

We present the perspectives of the different participants together by comparing similarities and differences according to their localities and their characteristics. Results were presented according to the categories of the social-ecological model [31] and the WHO building blocks [32]. Fig 2 summarises the findings according to the social-ecological model and the WHO building blocks.

### National level

#### Leadership and governance

The WHO building block [32] on leadership and governance considers the policies, regulations and institutions that oversee and guide the health system and ensure the efficient and equitable provision of health services.

Although screening of syphilis and HIV is part of the ANC protocol in Nepal, it is not available in all facilities, especially not in rural health posts. Hepatitis B screening is not covered by national guidelines and is available in a limited number of health facilities. This is despite claims to the contrary by the decision-maker interviewed from the Family Welfare Division of the Ministry of Health. *[PM2*: *HIV screening is conducted as a routine practice in Nepal*, *I remember it being implemented for a very long time*, *for more than 20 years*. *Hepatitis B and syphilis are also conducted routinely in every health institution in Nepal*.*]* The implementation of elements included in the national guidelines is left to the discretion of the local government at the municipal level. Health workers/officials have to convince their elected representatives to give them a budget for it. The services included in the ANC package and the charges are decided by hospital management committees and by the municipality in the case of health posts. In the context of Nepal’s federalization introduced in 2017, the role of provincial and local governments is still unclear. *[PM3*: *For a long time*, *it was not clear whether the central or the federal government should look over the health office of the districts*. *Finally*, *the Sthaniya Ain [Local Law] has been introduced*. *It clarifies the services that fall under the local government*. *However*, *it’s still confusing whose responsibility it is to procure materials for the provincial level*.*]*

#### Health budget and health financing

The financing building block (WHO 2007) concerns raising adequate funds for the healthcare system, allocating resources, and protecting people from the high costs of care-seeking.

The Nepalese health system is largely dependent on external donors. *[PM3*: *From what I know*, *the Government of Nepal has enough money for regular salaries*, *management and running some programs but the rest of the money is given by donors*.*]* The Ministry of Health and Population (MoHP) decides on health priorities based on consultation with its various bodies, and diplomatic and policy sectors. It discusses at the national level how health projects can be financed and whether it will be necessary to seek external funding to meet the needs.

The ANC protocol also states that any ANC-related examination should be provided free of charge. However, STI screening is not always available for free. *[HW7*: *When I was working in the maternity ward*, *there was a minimum charge*. *It was lower than in other hospitals*, *but it was not free*.*]* At the level of health facilities, municipalities make decisions for health posts regarding the budget allocated, the purchase of equipment such as test kits and the services to be provided free of charge. *[PM4*: *If it is a protocol guideline which is not mandated by the Constitution*, *then it is the discretion of the local government or the health coordinator to implement it*. *First*, *they need to be convinced that it is important*. *Then*, *they need to convince their elected members to give them a budget for implementation*. *Building a facility might be more appealing than just adding a small service which no one really knows*.*]* Public hospitals are funded by provincial governments. The management committee of each hospital has the power to decide and prioritise which services to include in the ANC package and which services to provide free of charge, based on the recommendations in the government protocols. It must coordinate with municipal public health officials and doctors to make a service mandatory.

ANC visits are free of charge at health posts. In public hospitals, the price varies from one institution to another and depends on the content of the examinations. *[HW7*: *ANC services costs in public hospitals are low but they depend on the programs that the government is running*.*]* Participants complained about the price of ANC visits in private hospitals. *[PW4*: *In private health facilities*, *they take a lot of money*, *so it is better to go to health posts or government hospitals*.*]* This difference in cost justifies participants’ preference for public health facilities over private ones. *[PW8*: *I did everything in a private hospital*. *I only came here [PMWH] three months ago because of monetary issues*. *It was too costly in the private hospital*.*]*

When available at health posts, blood tests are provided free of charge. As with ANC visits, the price of blood tests varies from one public hospital to another. Blood tests are more expensive in private facilities, and patients complain about this to doctors. *[HW7*: *In private hospitals*, *serology for hepatitis B is considered to be very expensive in comparison to HIV because it requires more equipment*. *Patients come and tell us*: *“these three tests are expensive*, *doctor”*.*]* It is common for patients to go to private facilities for ANC visits, but to go to public facilities for blood tests because they are cheaper. *[PW8*: *I was advised by the doctor that it’s costly in the private hospital*, *so I went to a laboratory in Baneshwor*.*]* Participants cited money as a reason for refusing blood screening during pregnancy. *[PW6*: *I have not done blood tests because I don’t have money*. *If I had money*, *then I would surely do the test*.*]*

Overall, healthcare professionals do not have a clear idea of the cost of the services they provide. *[I*: *What is the cost of a blood test for haemoglobin*, *HIV*, *syphilis and hepatitis B*? *Can you tell us the exact cost*? *HW6*: *No*, *I have no idea*.*]*

### Community-level

#### Taboo around sexual health

Most pregnant women, husbands and mothers-in-law were reluctant to talk about STIs. They laughed nervously, cut the discussion short or refused to answer questions. Also, the topics discussed were one of the main reasons why people refused to participate in the interview. This attitude of people not being ready to open up about the subject was also found in the interviews with health professionals.

Many interviewees pointed out that women may feel embarrassed to talk about sexual health, even with doctors. *[HW1*: *Even patients who come here cannot talk openly about their reproductive health problems*. *Sometimes*, *they come to us and secretly tell us their problems*.*]* Especially if the doctor is a male. *[PW2*: *It would be better if there was a female doctor instead of a male doctor*. *If there is a female doctor*, *then we can talk openly about our problems*.*]* This was often a reason given by participants for the need for the husband or someone to accompany the woman. *[H7*: *If there is a male doctor*, *then it can be difficult for the woman*. *The husband should go to make the situation easier*.*]*

#### Discriminatory attitudes and behaviours

Discriminatory behaviours regarding hepatitis B and syphilis were difficult to capture, as people were generally unaware of these particular diseases. However, the interviews revealed strong community stigma and peer pressure on HIV-positive people. *[H9*: *Some might feel the fear of embarrassment in society if the result comes positive because there is a practice of showing negative attitudes and discriminatory behaviour towards infected people in our society*.*]* HIV-positive people are often discriminated against and even excluded from society. *[HW2*: *The stigma here is that if you have contracted HIV*, *your life is over*. *How will I show my face*? *How am I going to get around*?*]* Thus, several participants emphasised the importance of awareness-raising programmes at the community level. *[PM1*: *Providing drugs is not enough*. *The main thing is to raise awareness*. *If people are aware*, *they can take care of themselves during pregnancy*.*]*

### Institutional level

#### Service delivery

The service delivery building block [32] focuses on providing equitable access to essential health services that meet the needs of the population by guaranteeing the availability of resources and the quality of the services provided.

*Availability of services*. The availability of blood tests depends on health facilities. Blood tests are done at the first ANC visit, usually after the third month of pregnancy. STIs are tested during pregnancy with other tests such as blood group or haemoglobin, using the same blood sample. The content of the blood tests depends on the healthcare facility. *[HW3*: *The test for HIV was considered important but not the ones for syphilis and hepatitis B*. *With time*, *the demand for other tests also gradually increased*. *Now*, *the ANC check-up package includes haematology*, *biochemistry*, *and serology tests*.*]*

Participants reported long waiting times in some cases, but this did not seem to discourage them from accessing ANC services. *[I*: *Sometimes it takes 2 or 3 hours to meet the doctor and sometimes even a whole day without eating any food*.*]* Waiting times for ANC visits, blood tests and results vary from one health facility to another. According to interviews, waiting times for the test can range from zero in some health posts to several hours in public hospitals for ANC visits, and from one hour in health posts or some provincial hospitals to several days in the PMWH to receive blood test results. Distance to health facilities did not appear to be an issue for blood testing. Participants interviewed in the PMWH sometimes reported coming from far away to access that particular hospital. *[H9*: *I came with my wife on a motorbike*. *It took around 2*.*5 hours*.*]*

Many interviewees referred, directly or indirectly, to the referral of patients from lower- to higher-level facilities. One example that emerged repeatedly in the interviews was the referral of patients from health posts that do not have laboratory or ultrasound facilities, or in case of complications during pregnancy, to public hospitals. *[HW4*: *We don’t have ultrasound facilities*. *If an anomaly is detected in time*, *we are obliged to refer them elsewhere*.*]* Patients are also referred from one department to another within the same hospital. Several problems with the referral system emerged from the interviews. It sometimes makes it difficult for people to access care, creates confusion and leads to loss of follow-up during pregnancy. *[HW4*: *They find it complicated when we refer them to the hospital*.*]* This also sometimes leads to the saturation of the largest public hospitals. *[HW4*: *Everyone knows PMWH*, *but it is crowded most of the time*.*]* This referral system is sometimes used as an excuse for not taking care of HIV-positive patients. *[HW2*: *They would not admit an HIV-positive patient*. *They would refer him to another health facility*.*]*

*Quality of care*. Overall, participants reported a high level of overcrowding in public hospitals that influence the quality of the services provided. *[PW12*: *There is so much crowd that patients enter the room for their turn before the previous one has completed the check-up*. *We can’t ask for anything and doctors don’t have time to explain things*.*]*

Even when blood tests are carried out, the quality of services is not always good. Some participants reported errors, receiving other people’s results or unreadable reports. *[PW11*: *When I had a miscarriage*, *I did a blood test in a polyclinic nearby and my blood group was different*. *Since then*, *I don’t want to do any tests there again because the report might have been exchanged with someone else*.*]* Others complained that the results of the blood tests were not explained to them by health workers. *[PW12*: *I came back after three days to get the report*, *but they did not tell me anything about it*. *I asked them but nobody answered*.*]*

Most of the participants said that they had not been counselled about blood tests and STIs. *[PW2*: *Health workers didn’t tell me anything*. *They just took the blood sample*. *It was very crowded*.*]* When counselling is provided, it is for HIV, but not for hepatitis B or syphilis. *[HW6*: *The counselling is about the prevention of HIV transmission from mother to child*. *I do not need to tell them about hepatitis B and syphilis*.*]* According to health workers, this depends on the health facility. For example, Tribhuvan University Teaching Hospital has a nurse who specialises in counselling, while health workers at other health facilities involved in ANC visits reported not doing counselling at all. Health workers justified not counselling pregnant women by the lack of time or space to do so, but also by the lack of interest or capacity to understand the patients. *[HW7*: *In most health facilities*, *there is limited time for counselling*. *We cannot give half an hour or an hour to a patient when we have to deal with 100 or 150 patients*. *Sometimes*, *when people hear about HIV*, *they become reluctant*, *so we have to explain it to them*. *For hepatitis B*, *if patients understand and are willing to listen*, *then we explain to them too*. *Counselling is not done uniformly between patients*, *sometimes the explanations are done correctly but sometimes they are not*.*]* Some health workers also felt that it was easier not to mention STIs, as this would lead women to refuse blood tests. *[HW1*: *The easiest way to counsel them about blood screening is to tell them that the test should be done to see if there is a problem in the blood*. *If we explain each test to them*, *they may not be able to understand it*. *Also*, *mentioning STIs may make them angry because they may feel insulted*. *They don’t readily accept such tests*.*]* However, most health workers who report not counselling patients also say that they are happy to answer women’s specific questions about blood tests. *[HW4*: *We tell them that we will do a blood test*. *If they ask*, *we tell them everything*, *but they never ask this kind of question*.*]*

Two health workers in the rural area cited language as a barrier to providing services to individuals, primarily counselling. *[HW1*: *At first*, *I was confronted with the problem of the Awadhi language*. *I’m still struggling to make sentences*, *but I can speak well enough to communicate*.*]*

#### Health workforce

The building block [32] that considers the health workforce addresses the availability, distribution, and capacity of healthcare workers.

*Training and shortage of health workers*. Most interviews revealed a shortage of healthcare workers, especially skilled workers. Many participants pointed to the large number of patients that need to be cared for by a limited number of health workers. *[H1*: *Thousands of people go to hospitals every day*, *so they are quite quick and give you information on the subject very quickly*.*]* This shortage results in a lack of time to provide health services and threatens their quality. *[PM4*: *Most of the ANC rooms are busy*. *There will be women inside the room and the process of ANC is less than 5 minutes*.*]* Participants also complained about the lack of follow-up by doctors. *[PW9*: *You want to show your report to the same doctor*, *but it doesn’t happen that way*. *Even if it’s the same day*, *the doctor changes from time to time*.*]*

Training of health workers is usually provided by the health facilities themselves or by the municipality, based on the MoHP recommendations and guidance. NGOs are also sometimes responsible for training health workers on specific topics. Some health workers reported that they had not received training for decades. *[HW6*: *After 2015*, *we did not receive any training*. *I had a 7-day training on the prevention of mother-to-child transmission of HIV and a 10-day training on breastfeeding*, *but it was about 8–9 years ago*.*]* Almost all complained about the lack of regular training. *[HW4*: *Nowadays*, *the municipality provides the training*, *but it is not enough to improve the skills like it used to be before*.*]*

*Attitudes of health workers*. Regarding the attitude of the health workers, most pregnant women and their relatives said that the health workers had the right attitude, often described as kind. *[PW7*: *They are always cheerful and behave decently with me*.*]* Some participants reported inappropriate behaviour by health workers, such as rudeness or hostility toward pregnant women. *[PW12*: *Sometimes*, *when we ask them about anything*, *they scold us so badly for not knowing it*. *Once*, *I asked them about where I could get the blood test reports from*. *They shouted at me so badly asking why I don’t read myself and whether I have eyes or not*. *I felt very bad*. *Some are good but some of them are very aggressive*.*]* Also, some participants complained about the lack of listening by health workers. *[I*: *Have you ever experienced someone who behaved badly with you*? *PW5*: *Yes*, *sometimes they do not listen to you at all*.*]* Concerning STIs, health professionals were generally reluctant to talk about discriminatory behaviour in their profession. However, some decision-makers and health workers revealed that this type of behaviour is quite common. *[HW2*: *Health workers still act rudely sometimes*. *Even HIV-positive people with health insurance are reluctant to come because the health staff do not treat them properly and send them from one place to another*.*]* They also indicated that the situation had improved. All health workers reported that they strive to maintain confidentiality, but face difficulties in practice, mainly due to lack of space.

The interviews revealed that, overall, people trust health workers. They often indicated that they give them good suggestions. *[ML3*: *Health workers in the health post will give her good suggestions*.*]* Participants said they were willing to listen to them and do what they recommended because they had better knowledge. *[ML2*: *We are uneducated*, *and we do not understand anything*. *If it affects the health of a pregnant woman as well as the child then*, *we will follow the doctor’s suggestion*. *We will try to follow whatever they tell us*.*]* However, some people suggested that people sometimes did not trust them and preferred to seek confirmation at different health facilities. *[PW2*: *The doctor said that there was no heartbeat*. *I did not believe it*. *The next day*, *I went to Bhairahawa for an ultrasound*. *They said the same thing there too*.*]*

#### Health information systems

The health information building block [32] involves the collection, analysis and dissemination of data and information relating to health to facilitate decision-making and monitor the health of populations.

In Nepal, women’s maternity data is only reported after three months of pregnancy. Any event or illness occurring before the third month of pregnancy is not recorded in the Integrated Health Management Information System (IHMIS). The recording of STI data is done manually by health workers in all health facilities. While HIV data seems to be systematically recorded, this is not the case for syphilis and hepatitis B. According to some health workers, the lack of available information on syphilis and hepatitis B is due to the lack of available test kits and the lack of staff and time to record the data. *[HW7*: *In all facilities*, *data is reported manually*. *Patient loads are high*, *outpatient departments are busy with sometimes 200 to 300 patients coming and manpower is limited*. *We try to record data as much as possible*, *but we do not write the data both on the patients’ cards and in our data*.*]*

#### Medical products

The building block [32] that considers access to essential medicines addresses the availability and appropriate use of medicines, vaccines, and medical equipment, with a focus on quality, safety, and affordability.

Interviews revealed a general lack of resources. *[PW9*: *They told me there was no heartbeat*. *They advised me to get admitted but due to a lack of beds at the emergency service*, *they could not admit me*.*]* While HIV testing kits are generally available in health facilities, syphilis and hepatitis B testing kits are often not available due to shortages. *[HW3*: *HIV test kits are usually available but not kits to test hepatitis B and syphilis*. *They are not funded by any program*, *and we cannot afford them*. *HIV kits are available through external funding*.*]* Laboratory services are often not available at health posts. *[PM4*: *We have ANC all over Nepal but screening for STIs is available only wherever there are laboratory facilities*. *That will be less than 200 government health facilities*, *hospitals*, *and a few Primary Health Care centres*. *The availability of ANC is very good in Nepal but when you go deep into the service and look at the quality and the components*. *Then what we call availability becomes poor*.*]* Many health workers also complained about the lack of appropriate counselling spaces. *[PM2*: *There are counselling rooms in the hospitals*, *but not in small health posts*.*]*

### Interpersonal level

#### Husband’s willingness to support

Overall, the husbands interviewed expressed a willingness to be involved in their wives’ pregnancies and often attested to supporting their wives with daily tasks. *[I*: *Do you support your wife during her pregnancy*? *H4*: *Yes*, *I support her 100%*. *I do not let her carry heavy objects or wash clothes*. *I encourage her to rest properly and follow doctors’ advice*.*]* However, some husbands reported not having any information about their wife’s pregnancy other than that the baby is doing well. *[H2*: *She said that the baby is normal and healthy*, *so I didn’t ask her about anything else*. *We were more concerned about the baby*, *so I didn’t think it was important to ask about anything else*.*]* While some couples discuss the pregnancy openly, others do not discuss it at all with each other. *[H4*: *We spontaneously exchange health-related information*, *such as what we should do*, *whether we should go for a walk*, *etc*.*]*

In urban settings, some husbands reported accompanying their wives on ANC visits. However, in large urban hospitals, they are sometimes not allowed to enter the room for check-ups, even if they want to *[H6*: *Health workers should welcome both the husband and his wife*. *They should tell me about the problems of my wife and that I should not worry*.*]* Husbands often expressed their inability to be there as much as they would like to be for their wives because of their work and the pressure to bring money into the household. *[H6*: *Sometimes when I did not have time to go with her due to office obligations*, *she went alone*. *Otherwise*, *we always go together*.*]*

In the migratory context of Nepal, among the eighteen families interviewed, four husbands, living in Kapilvastu, were reported not living with their wives now or in the previous months but abroad or in another city. Communication difficulties within the couple are sometimes reinforced by the lack of proximity when the husband has to migrate. In this situation, communication sometimes takes place through the pregnant woman’s in-laws. *[PW1*: *I told my sister-in-law first*, *and she shared it with everyone else*. *I*: *Didn’t you share the news with others*? *PW1*: *No because I was feeling shy*.*]*

For STIs in particular, in response to the hypothetical scenario presented, most husbands expressed that they would support their wives if they had symptoms of an STI. *[H7*: *If women get infected with STIs*, *they need full support from their husbands*.*]* Mainly if she needs their help in discussing her situation with health professionals. *[H3*: *Some women might feel shy*. *I will tell my wife that I will come with her*. *She should not be scared*.*]* However, several women and health professionals interviewed pointed out that in practice, some husbands are not as supportive and understanding. *[PW11*: *Although my husband is understanding*, *many pregnant women have husbands who do not understand or care about them*.*]* Most interviewees said that the husband should be the first to know about his wife’s sexual health problems and that husbands should also be tested as part of antenatal screening for STI. *[PW3*: *It is important and necessary that the husband take a blood test too*. *It will help to identify who is infected and how we can prevent it*.*]*

#### In-laws’ involvement

In joint households, which are very common in rural areas, the parents-in-law are involved in the pregnant woman’s health, mainly the mothers-in-law or sisters-in-law. Indeed, the topics of pregnancy, sexual health or STIs are more easily discussed with people of the same sex. *[I*: *Do you think she should tell her husband*? *PW1*: *Yes*, *and her mother-in-law or sister-in-law*, *but not her father-in-law*.*]* The involvement of in-laws may take the form of advice, pressure, or accompaniment during check-ups. *[HW3*: *If they are having repeated infections or lower abdominal pain during intercourse*, *they can’t share such things in front of their mother-in-law*. *The mother-in-law may try to normalise the problems and demoralise the patients by not giving importance to their problems*.*]* In contrast, some mothers-in-law support their daughters-in-law. *[ML1*: *It is normal to help my daughter-in-law like this*. *She left her father and mother and came to live with us as if we were her parents*. *We must help her and understand her pain*.*]* Pregnant women generally need the permission of their in-laws before making decisions about their health. *[I*: *If the doctor says that they need to take your blood for STI tests*, *what would you do*? *PW3*: *I will say that I will decide after consulting with my family*. *I will ask my relatives if they want it or not*. *I will do as per their wish*. *I can’t just do whatever I want*.*]* In the context of migration, the husband often entrusts his wife to his family. *[H1*: *Our family lives together*, *so even if I can’t help and support her*, *my family supports her when I’m not there*.*]*

#### Neighbours’ involvement

In rural settings, neighbours are also an important source of information about health in general, the availability of health services and pregnancy in particular. *[PW5*: *My neighbour told me to go for a check-up if I had any health problems*.*]* In the case of STIs, participants shared that they learned a person’s STI status from neighbours. *[I*: *Have you ever heard of anyone infected with HIV*, *syphilis*, *or hepatitis B*? *PW12*: *My neighbours said that someone has such a disease so we should not visit him*, *otherwise we would get infected too*.*]* They play an important role in spreading rumours about the health of individuals and stigmatising behaviour. *[H2*: *There was a rumour in the village that he has HIV*. *We cannot go to visit him after knowing this about him*.*]* This type of behaviour seemed to be more present in rural areas and was not reported by participants in the Kathmandu Valley. *[H1*: *In urban areas like Pokhara*, *everyone is busy with their own work*, *why should I interfere in other people’s life*?*]*

### Individual-level

#### Health negligence

Some participants reported that they follow health workers’ recommendations carefully, go for regular check-ups, and comply with treatments. *[PW1*: *I usually go for ultrasounds when they call me*. *I’ve never missed an appointment*.*]* On the contrary, some participants, through the description of certain situations or more directly the health workers interviewed, revealed the negligence of individuals in health-related matters. *[PW4*: *The doctor told me to visit again but I did not*.*]* In the case of family members and given their involvement in the pregnancies described above in rural settings, their neglect has direct consequences since women’s health and consultations depend on them. *[PW2*: *I asked my in-laws to go for another check-up because it might be a problem with medication*, *but they did not take me and I stopped the medicines*.*]*

Another established norm in Nepal that emerged from the interviews is that if there are no significant symptoms, people believe that there is no disease or that it will heal on its own or with home remedies. *[HW3*: *They first follow all the possible home remedies they can*. *If nothing works*, *then only they come to the hospital for help*.*]* Thus, people only go to the hospital if the illness is severe, and the symptoms are important. *[HW2*: *Patients only go to the hospital when their condition is really serious*. *For example*, *with STIs*, *if patients have severe back pain or abnormal discharge or something serious*, *then only they feel the need to go to the hospital*.*]* People also tend to hide their health problems in general especially when it comes to sexual health. *[PW11*: *Usually*, *people keep their disease secret*, *mostly in the village areas*. *It is similar in the case of miscarriage*. *For example*, *I did not reveal mine*.*]*

#### Knowledge about blood tests and STIs

When asked about their knowledge of ANC check-ups and blood tests, most participants were aware of their importance, although they did not know what was diagnosed by blood tests. When asked about their knowledge of STIs, most participants said they had heard of HIV and had some vague knowledge of the mode of transmission or symptoms. A limited number of educated participants have a good knowledge of the subject, mostly in urban settings. *[H9*: *HIV is a disease which gets transmitted through blood and sexual activities*. *It can get transmitted through blood by the use of needles*, *razors and sharp instruments if they are not sterilised*.*]* However, there is generally some confusion and misconception. *[H2*: *HIV can be transmitted by sitting and talking together*. *It can also be transmitted through sex or by living and sleeping together*. *If someone has this disease*, *we should not sit or live with him*. *We should keep our distance from such people*.*]* Participants reported hearing about it in the media, primarily on the radio, or during their studies. Most participants had never heard of syphilis and hepatitis B and had very low knowledge and awareness of their symptoms and mode of transmission.

#### Acceptance of blood tests

Consent for blood screening during pregnancy is given verbally by pregnant women. Health workers reported that blood tests were generally well accepted. This was confirmed by the pregnant women, husbands and mothers-in-law interviewed. The main reason given for the acceptance of blood tests was their importance to the health of the child and the mother. *[ML2*: *I would strongly advise the pregnant women to take a blood test timely as it helps to know if the health of the baby and mother is going well*.*]* Most of the people interviewed said they would accept the blood test, regardless of its purpose. This view was not shared by health professionals, who felt that most people would refuse it if it was explicitly explained to them that it was to test for STIs. *[HW2*: *If you organise a campaign to test blood for dengue*, *diabetes and malaria*, *they will readily agree to take the blood test*. *However*, *they will hardly agree to the same blood test for STIs*. *They would rather run away or scold you*.*]* Potential reasons for reluctance to accept blood tests that emerged from the interviews were financial constraints, but also the fact that people do not consider them necessary because they are healthy or, conversely, they fear the results. *[H1*: *There are many reasons for not agreeing to have a blood test*: *for fear of what people might say*, *for fear of the disease*, *and in some cases*, *some people may feel that it is not necessary*.*]*

#### Deciding which health facility to visit

People reported preferring to receive all their care in a single establishment. [*HW4*: *Patients feel that they would feel more comfortable if all services were provided in this health facility*, *including ultrasound*, *instead of having to go to hospitals*.] However, the decision to change health facilities during pregnancy sometimes comes from the individuals themselves. In rural areas, people sometimes come to the health post with reports of test results from private or public hospitals to confirm interpretations. This shows people’s trust in local health structures. *[H9*: *We took with us the report of the blood test we did at Binayak Hospital two or three days ago at the health post and they gave us some suggestions*.*]* In the Kathmandu Valley, people sometimes choose health facilities where to access ANC services, knowing that they will eventually be referred by health professionals. *[PW9*: *We thought that even if I go to a private hospital*, *they will refer me to this place if any complication arises*, *so why not come here directly*.*]* Participants also indicated that they went to larger public facilities, believing that the services provided would be of better quality, or to private clinics to avoid the crowds in public hospitals. *[HW6*: *People from elsewhere think that they have to come to this hospital to deliver properly and avoid complications during operations*. *They may think that there are few or fewer qualified doctors in their area*.*]* In the latter case, people usually come for a few ANC visits but cannot afford to do them all. *[HW7*: *Per ANC visit*, *it costs 500 rupees*, *so they do one visit here and then we lose the follow-up*. *They think something like let’s pay for once to deviate from the government hospital line*.*]*

### Differences and similarities in perceptions between respondents in Kapilvastu (rural plains) and Kathmandu (urban)

Rural and urban settings differed in terms of respondents’ perceptions and the barriers and facilitators to antenatal screening identified. While, on the whole, the lack of resources in the health system emerged as one of the main barriers to antenatal screening in both urban and rural areas, the lack of STI screening services was reported more by participants in Kapilvastu, mainly at health posts. Similarly, referral of patients from health posts to public hospitals with laboratories was more common in rural areas. All health workers said that they tried to maintain confidentiality, but health workers working in Kathmandu said that they encountered difficulties in doing so, mainly due to lack of space. In Kathmandu, many health workers also complained about the lack of appropriate counselling facilities. This issue did not come up in the Kapilvastu interviews. In urban areas, participants highlighted the large number of patients who have to be cared for by a limited number of health workers, mainly in large urban hospitals, and participants complained about waiting times and husbands not being allowed to enter the room with their wives for examinations, even if they wanted to.

While husbands in both rural and urban areas expressed their inability to be as present as they would like for their wives, the reasons cited differed between the two settings. In Kathmandu, the main reason was work. In rural areas, husbands often lived abroad or in another town. In rural areas, joint households are more common than in Kathmandu, which makes the involvement of in-laws in the pregnant woman’s health more important there. Similarly, the involvement of neighbours as a source of information, the spreading of rumours about people’s health and stigmatising behaviour were not reported in the Kathmandu valley, whereas they were by several participants in Kapilvastu. While the attitude of people not being ready to open up on the subject of STIs was found in both rural and urban areas, this finding was more exacerbated among participants in Kapilvastu. Some health workers in Kapilvastu were not even prepared to say the words "sexually transmitted diseases".

### Differences and similarities in perceptions between different household members

In interviews with the members of the same households some interesting similarities and differences between household members’ perceptions emerged. Husbands and mothers-in-law were generally less reluctant to talk about pregnancy and their knowledge of STIs than pregnant women. Pregnant women were particularly reluctant to talk about the subject, even if the interviewer was of the same sex. In the joint households interviewed in Kapilvastu, most of the pregnant women were embarrassed to talk about their mother-in-law’s involvement in their pregnancy, even if she was not in the room, whereas the mothers-in-law were not embarrassed to talk about their daughter-in-law.

Husbands expressed support for their wives if they had STI symptoms, while pregnant women and mothers-in-law tended to perceive husbands’ support as weak in some cases, as husbands were not as understanding as they might say. The main reason given by husbands for accepting blood tests was their importance for the child’s health, while the health of both mother and child was mentioned by mothers-in-law and pregnant women. Husbands and pregnant women agreed that they wanted the husbands to be involved in their wives’ pregnancies but tended to differ in their perceptions of in-laws’ involvement. Some pregnant women complained about the involvement of their mothers-in-law and sisters-in-law in their pregnancy, particularly in Kapilvastu, while husbands and mothers-in-law were quite satisfied with it.

## Discussion

This qualitative study showed that antenatal screening for HIV, syphilis and hepatitis B in Nepal involved many stakeholders and was influenced by factors at different levels of the socioecological model and across the different WHO building blocks. We identified implementation gaps in service delivery, health workforce, health information system, medical products, health financing and governance and leadership. Interpersonal and individual factors, such as the involvement of husbands and in-laws in health decisions and people’s knowledge of STIs, play a key role in the uptake of antenatal screening.

At the national level, different points need to be discussed regarding the leadership and governance and health financing building blocks. Syphilis and HIV screening is part of the ANC protocol in Nepal [11] and is implemented by the Ministry of Health and Population with support from various international organisations, including UNICEF, WHO, and the United States Agency for International Development (USAID). Nepal has adopted an opt-out screening strategy, similar to that of Thailand [36] and Sri Lanka [37], which have eliminated vertical transmission. However, in practice, we found that women were not really offered the choice to opt-out as they were unaware of the purpose of the blood test. On the other hand, hepatitis B screening is not covered by national guidelines.

Despite an overall lack of clarity in roles and responsibilities between different government levels since the federalisation in 2017 [38], local authorities play a key role in implementing health protocols and allocating funding for antenatal screening. Yet the elected officials often lack the technical training or experience needed to make informed decisions [39] and antenatal screening for STIs is often not a priority [16, 39]. Meanwhile, federalisation has added layers of decision-making, increasing administrative burdens and causing delays in bringing about change [39]. In addition, local priorities have shifted from social sectors, including health, to visible projects such as road and building construction, which are seen as beneficial for re-election [39, 40]. Political instability also makes it difficult to implement long-term decisions [41].

At the community level, a lack of knowledge and misconceptions about HIV transmission results in a high level of stigmatisation of HIV-positive people. Consistent with other studies [42, 43], we found a significant taboo surrounding sexual health and HIV, leading to discriminatory behaviour. Although our questions addressed the stigma attached to STIs, discussions predominantly focused on HIV, thus limiting information on the stigma associated with syphilis and hepatitis B. We found that Nepalese people often delay seeking care, especially for STIs, due to shame and fear of judgement.

At the institutional level, different WHO building blocks were investigated. Implementation of HIV screening is limited and uneven across regions, with 25% of pregnant women in Gandaki Province receiving an HIV test during ANC compared to only 7% in Karnali Province [44]. Facilities are less well equipped to provide syphilis screening than HIV with only 22% of facilities having the equipment to screen for syphilis [45], despite national recommendations, indicating incomplete integration of these services [16]. WHO recommends the dual HIV/syphilis test in ANC settings for its cost-effectiveness, reduced loss to follow-up, and increased syphilis testing coverage [46]. These tests are available for less than USD 1 each and require minimal training and lab infrastructure [47]. Similar rapid tests are available for hepatitis B. Based on integrated rapid-test approaches, Thailand’s model has been recognised by WHO as having reached the elimination targets for the vertical transmission of HIV and syphilis [36]. A similar rapid-test approach should be prioritised in Nepal to facilitate the availability of antenatal screening in health facilities and increase screening uptake [48].

HIV counselling reduces stigma and improves community knowledge [49]. In Nepal, however, counselling is not uniform, and women are often not informed of the purpose of their blood tests. Reducing stigma requires community awareness campaigns and a culture of transparency on the part of health professionals regarding the objectives of screening and the sharing of results. Lack of communication by healthcare professionals can perpetuate stigma. The more healthcare professionals contribute to stigma by avoiding discussions about diseases, the slower the resolution of stigmatisation will be.

We found that referrals for antenatal care and antenatal blood tests are common in Nepal, particularly in rural areas where access to screening services is limited [45]. Although referrals are intended to provide timely care, they can overload hospitals with cases that do not require specialist attention. The high level of trust in local health facilities suggests that there is scope to improve screening and counselling at health posts, reducing the need for referrals. Significant investment in lower-level public health laboratories is required for effective triple screening.

Regarding the health workforce building block, the shortage of qualified healthcare staff lengthens waiting times and threatens the quality of services. In addition, healthcare staff often lack adequate training on STIs, which is essential for reducing stigmatising behaviour [50, 51]. These problems have also been identified in studies elsewhere in Nepal such as the mountain area of Jumla [52] and the southern plains [53].

Of the three diseases considered, HIV has benefited from more screening resources, awareness campaigns and media coverage, due to the global interest and the substantial international financial support it receives [54]. On the other hand, syphilis and hepatitis B, which are more prevalent in Nepal, lack resources and systematic data. Hepatitis B screening and prevalence are excluded from IHMIS and national surveys [55], while syphilis, although included, is not systematically reported. This hampers monitoring [56], leading to neglect of these diseases and their vertical transmission. A robust national monitoring and evaluation system, which uses data to improve programmes at national and sub-national levels, has played a crucial role in Thailand’s success in eliminating vertical transmission of HIV and syphilis [36].

At the interpersonal level, we found that the husband’s lack of involvement or support for antenatal screening, and pregnancy in general, is often due to a lack of time, caused by the pressure to earn money, rather than an intention to withhold support. Husbands’ lack of engagement is reinforced by migration, especially in rural areas [20]. In Nepal, women usually move into their husband’s households after marriage [57, 58], where their decision-making power is often weak [59, 60]. This is changing over recent years with people no longer living in joint families, especially in urban contexts. However, social position within the household remains an important determinant of decision-making capacity in Nepal [61, 62]. The migration of husbands often complicates the communication between spouses and increases the involvement of in-laws in women’s pregnancies. We found that women in joint households generally need the approval of their in-laws before making decisions about their health, which has also been found by others [63, 64].

Husbands expressed that they would provide support to their wives in the hypothetical scenario of positive STI test results, but this was not always confirmed in interviews with women and health workers. This discrepancy might indicate underlying issues such as potential interpersonal violence, which may prevent women from expressing themselves openly or asking for help. Although not raised in our interviews, similar concerns have been raised in other countries in the region, such as India [65] or Vietnam [66].

At the individual level, the priority given to HIV over other diseases is reflected in people’s knowledge. We found relatively good knowledge about HIV but very little about syphilis and hepatitis B. Our findings underscore the importance of awareness programs at the community level coupled with increased resources for individual counselling by health workers.

Financial barriers in households hinder access to and acceptance of antenatal screening, a problem identified in other Asian studies [67, 68]. As well as to get blood test screening and ultrasound services, people move from one facility to another to save money, leading to long waits and overcrowding in public hospitals, disrupting the continuity of care. The choice of healthcare facility is influenced by price disparities, perceived differences in quality between public and private facilities [69, 70], recommendations from neighbours or family members [71, 72] and availability of services such as blood tests and ultrasound.

Our study has several limitations. Firstly, in Kathmandu, participants were recruited through the PMWH, which meant that all the women interviewed had already attended at least one ANC visit and we were not able to access women who had not received ANC. Secondly, given the sensitivity of the subject, people were sometimes reluctant to answer questions about STIs. When people said they did not know about HIV or how it was transmitted, it was almost impossible to determine whether this was a real lack of knowledge or shyness about discussing the subject. Although we planned to interview husbands, mothers-in-law, and pregnant women from the same households, this was only possible in a subset of households in Kapilvastu and not at all in Kathmandu. This limited our capacity to triangulate and compare perceptions of different members within households. The local word for syphilis was not always used by the data collectors during interviews in Kapilvastu, which caused confusion and limited how much we could infer about syphilis from these interviews. However, even in interviews where the local terms were used knowledge of syphilis was minimal.

Despite limitations, our study offers unique insights into factors influencing integrated antenatal screening for HIV, syphilis, and hepatitis B in Nepal. Improving integrated antenatal screening will require a multi-sectoral approach that involves strengthening healthcare systems, addressing stigma and discrimination, and increasing investment in syphilis and hepatitis B prevention programs. It will require greater engagement with communities through awareness programs and enhancement of the role of health posts, especially in big cities, to regulate the flow of patients in large public hospitals. Decongestion of the public health system might also be facilitated by the regulation of prices for services in the private sector. Based on other countries’ successful opt-out approach, opt-out hepatitis B screening should be integrated into the ANC package. Rapid tests should also be encouraged in Nepal.

## Conclusion

Our study highlights the multi-level challenges of integrating antenatal screening for HIV, syphilis and hepatitis B in Nepal. We identified gaps in different WHO building blocks including service delivery, health workforce, health information, medical products, health financing and governance and leadership. We also found that interconnected factors played an important role at different levels of the socio-ecological model. Local authorities play a key role but often lack technical expertise, and community stigma remains a significant barrier. Improving the availability of screening by using rapid tests, investing in strengthening health systems and implementing an opt-out approach to hepatitis B screening, similar to that for HIV and syphilis, are crucial steps. Effective community engagement through awareness campaigns to reduce stigmatisation and investment in lower-level public health facilities is essential to improve the uptake of screening and reduce vertical transmission of these infections.

## Supporting information

S1 Checklist Inclusivity in global research. (DOCX)

S1 File Vignette 1—A pregnant friend heard about ANC visits. (DOCX)

S2 File SRQR guidelines checklist. (DOC)

## References

[1] UNAIDS. Country progress report—Nepal, Global AIDS Monitoring. 2020.

[2] Ministry of Health and Population. National Strategy for Viral Hepatitis B and C. 2023.

[3] Upreti P. Prevalence of STIs among nepalese women population. Sexually Transmitted Infections. 2019;95: A164.

[4] Shakya S, Thingulstad S, Syversen U, Nordbø SA, Madhup S, Vaidya K, et al. Prevalence of sexually transmitted infections among married women in rural Nepal. Infectious diseases in obstetrics and gynecology. 2018;2018. doi: 10.1155/2018/4980396 30224859 PMC6129351

[5] Dao H, Mofenson LM, Ekpini R, Gilks CF, Barnhart M, Bolu O, et al. International recommendations on antiretroviral drugs for treatment of HIV-infected women and prevention of mother-to-child HIV transmission in resource-limited settings: 2006 update. American journal of obstetrics and gynecology. 2007;197: S42–S55. doi: 10.1016/j.ajog.2007.03.001 17825650

[6] Wendel GD Jr, Sheffield JS, Hollier LM, Hill JB, Ramsey PS, Sánchez PJ. Treatment of syphilis in pregnancy and prevention of congenital syphilis. Clinical Infectious Diseases. 2002;35: S200–S209. doi: 10.1086/342108 12353207

[7] van Zonneveld M, Van Nunen A, Niesters H, De Man R, Schalm S, Janssen H. Lamivudine treatment during pregnancy to prevent perinatal transmission of hepatitis B virus infection. Journal of viral hepatitis. 2003;10: 294–297. doi: 10.1046/j.1365-2893.2003.00440.x 12823596

[8] UNAIDS. AIDSinfo. Geneva: Joint United Nations Programme on HIV/AIDS. 2018. Available: http://aidsinfo. unaids.org/

[9] WHO. Regional framework for the triple elimination of mother-to-child transmission of HIV, hepatitis B and syphilis in Asia and the Pacific, 2018–2030. 2018.

[10] Ministry of Health and Population. National HIV Testing and Treatment Guidelines 2017. 2017.

[11] Ministry of Health and Population. Nepal Safe Motherhood and Newborn Health Road Map 2030. 2019.

[12] WHO. Elimination of Mother-to-Child Transmission HIV, Hepatitis B and Syphilis in Asia and the Pacific. 2024. Available: https://www.aidsdatahub.org/thematic-areas/emtct/triple-emtct-data

[13] Trivedi S, Taylor M, Kamb ML, Chou D. Evaluating coverage of maternal syphilis screening and treatment within antenatal care to guide service improvements for prevention of congenital syphilis in Countdown 2030 Countries. Journal of Global Health. 2020;10. doi: 10.7189/jogh.10.010504 32280458 PMC7138526

[14] Pokharel S, Abbas H, Ghimire A. Response to antenatal HIV testing and prevention of parent to child transmission: an experience in a tertiary hospital in Chitwan, Nepal. Journal of College of Medical Sciences-Nepal. 2011;7: 36–38.

[15] Thapa R, Yang Y, Kang JH, Nho J-H. Empowerment as a predictor of HIV testing among married women in Nepal. Journal of the Association of Nurses in AIDS Care. 2019;30: 563–574. doi: 10.1097/JNC.0000000000000021 31461738

[16] Acharya K, Thapa R, Bhattarai N, Bam K, Shrestha B. Availability and readiness to provide sexually transmitted infections and HIV testing and counselling services in Nepal: evidence from comprehensive health facility survey. BMJ open. 2020;10: e040918. doi: 10.1136/bmjopen-2020-040918 33323441 PMC7745329

[17] Ministry of Foreign Affairs. Nepal Profile–Ministry of Foreign Affairs Nepal MOFA. 2023 [cited 30 May 2023]. Available: https://mofa.gov.np/nepal-profile-updated/

[18] Ministry of Health and Population. Health Management Information System (HMIS) Portal Website. 2023 [cited 30 May 2023]. Available:: http://hmis.mohp.gov.np/portal/apps/healthstats/

[19] UNDP, Government of Nepal. Nepal Human Development Report. Kathmandu: Government of Nepal, National Planning Commission. 2014.

[20] International Organization for Migration. Migration in Nepal: a Country Profile 2019. 2019.

[21] National Statistics Office. Preliminary Report of National Population Census 2021. 2021. Available: https://censusnepal.cbs.gov.np/Home/Details?tpid=1

[22] Ministry of Education, UNESCO, UNICEF. Global Initiative on Out-of-School-Children–Nepal Country Study. 2016.

[23] Elmir R, Schmied V, Jackson D, Wilkes L. Interviewing people about potentially sensitive topics. Nurse researcher. 2011;19. doi: 10.7748/nr2011.10.19.1.12.c8766 22128582

[24] Dempsey L, Dowling M, Larkin P, Murphy K. Sensitive interviewing in qualitative research. Research in nursing & health. 2016;39: 480–490. doi: 10.1002/nur.21743 27434172

[25] Gram L, Skordis-Worrall J, Mannell J, Manandhar DS, Saville N, Morrison J. Revisiting the patriarchal bargain: The intergenerational power dynamics of household money management in rural Nepal. World Development. 2018;112: 193–204. doi: 10.1016/j.worlddev.2018.08.002 30510348 PMC6167740

[26] Marshall B, Cardon P, Poddar A, Fontenot R. Does sample size matter in qualitative research?: A review of qualitative interviews in IS research. Journal of computer information systems. 2013;54: 11–22.

[27] Barter C, Renold E. The use of vignettes in qualitative research. Social research update. 1999;25: 1–6.

[28] Törrönen J. Using vignettes in qualitative interviews as clues, microcosms or provokers. Qualitative Research Journal. 2018;18: 276–286.

[29] Groot T de, Jacquet W, Backer FD, Peters R, Meurs P. Using visual vignettes to explore sensitive topics: a research note on exploring attitudes towards people with albinism in Tanzania. International Journal of Social Research Methodology. 2020;23: 749–755.

[30] Green J, Thorogood N. Qualitative methods for health research. sage; 2018.

[31] McLeroy KR, Bibeau D, Steckler A, Glanz K. An ecological perspective on health promotion programs. Health education quarterly. 1988;15: 351–377. doi: 10.1177/109019818801500401 3068205

[32] WHO. Strengthening health systems to improve health outcomes: WHO’s framework for action. Geneva: WHO. 2007.

[33] Ritchie J, Spencer L, Bryman A, Burgess RG. Analysing qualitative data. Routledge, London; 1994.

[34] Tong A, Sainsbury P, Craig J. Consolidated criteria for reporting qualitative research (COREQ): a 32-item checklist for interviews and focus groups. International journal for quality in health care. 2007;19: 349–357. doi: 10.1093/intqhc/mzm042 17872937

[35] O’Brien BC, Harris IB, Beckman TJ, Reed DA, Cook DA. Standards for reporting qualitative research: a synthesis of recommendations. Academic medicine. 2014;89: 1245–1251. doi: 10.1097/ACM.0000000000000388 24979285

[36] Sidibé M, Singh PK. Thailand eliminates mother-to-child transmission of HIV and syphilis. 2016;387: 2488–2489. doi: 10.1016/S0140-6736(16)30787-5 27291996

[37] WHO. Country Report Sri Lanka Validation of Elimination of Mother-to-child Transmission of HIV and Syphilis. 2019.

[38] Acharya KK, Scott J. A study of the capabilities and limitations of local governments in providing community services in Nepal. Public Administration and Policy. 2022;25: 64–77.

[39] Thapa R, Bam K, Tiwari P, Sinha TK, Dahal S. Implementing federalism in the health system of Nepal: opportunities and challenges. International journal of health policy and management. 2019;8: 195. doi: 10.15171/ijhpm.2018.121 31050964 PMC6499910

[40] Acharya KK. Federalism practice in Nepal: Does it move in the expected course. Dhaulagiri Journal of Sociology and Anthropology. 2021;15: 20–34.

[41] Analytica O. Government instability will be endemic in Nepal. Emerald Expert Briefings. 2023.

[42] Chaudhary S, Kakchapati S. Social stigma, discrimination, and their determinants among people living with HIV and AIDS in Sudurpashchim Province, Nepal. HIV & AIDS Review International Journal of HIV-Related Problems. 2022;21: 230–238.

[43] Bhattarai N, Bam K, Acharya K, Thapa R, Shrestha B. Factors associated with HIV testing and counselling services among women and men in Nepal: a cross-sectional study using data from a nationally representative survey. BMJ open. 2021;11: e049415. doi: 10.1136/bmjopen-2021-049415 34862281 PMC8647541

[44] UNICEF. Nepal MICS Statistical Snapshot. 2019.

[45] Ministry of Health and Population, New ERA, ICF. Nepal Health Facility Survey 2021. Kathmandu, Nepal, Rockville, Maryland, USA: Ministry of Health and Population, New ERA and ICF; 2022. Available: https://www.dhsprogram.com/pubs/pdf/SPA35/SPA35.pdf

[46] WHO. Dual HIV/syphilis rapid diagnostic tests. 2023.

[47] Global Fund. Pooled Procurement Mechanism Reference Pricing. 2024.

[48] Cohn J, Owiredu MN, Taylor MM, Easterbrook P, Lesi O, Francoise B, et al. Eliminating mother-to-child transmission of human immunodeficiency virus, syphilis and hepatitis B in sub-Saharan Africa. Bulletin of the World Health Organisation. 2021;99: 287. doi: 10.2471/BLT.20.272559 33953446 PMC8085625

[49] Nyblade L, Stangl A, Weiss E, Ashburn K. Combating HIV stigma in health care settings: what works? Journal of the international AIDS Society. 2009;12: 1–7. doi: 10.1186/1758-2652-12-15 19660113 PMC2731724

[50] Bohren MA, Vazquez Corona M, Odiase OJ, Wilson AN, Sudhinaraset M, Diamond-Smith N, et al. Strategies to reduce stigma and discrimination in sexual and reproductive healthcare settings: A mixed-methods systematic review. PLOS Global Public Health. 2022;2: e0000582. doi: 10.1371/journal.pgph.0000582 36962453 PMC10021469

[51] WHO. Progress report on the regional action plan for viral hepatitis in the WHO South-East Asia Region (2016–2021). 2020.

[52] Tamang P, Simkhada P, Bissell P, van Teijlingen E, Khatri R, Stephenson J. Health facility preparedness of maternal and neonatal health services: a survey in Jumla, Nepal. BMC health services research. 2021;21: 1–10. doi: 10.1186/s12913-020-05996-8 34583697 PMC8479916

[53] Lama TP, Munos MK, Katz J, Khatry SK, LeClerq SC, Mullany LC. Assessment of facility and health worker readiness to provide quality antenatal, intrapartum and postpartum care in rural Southern Nepal. BMC health services research. 2020;20: 1–12. doi: 10.1186/s12913-019-4871-x 31906938 PMC6945781

[54] The Global Fund. The Global Fund Data: Nepal Overview. 6 Jun 2023. Available: https://data.theglobalfund.org/location/NPL/overview

[55] WHO. Report on global sexually transmitted infection surveillance 2018. World Health Organization; 2018.

[56] Ministry of Health and Population. DHIS2 Tracker, NHIS Nepal. 2010.

[57] Pearlman J, Pearce LD, Ghimire DJ, Bhandari P, Hargrove T. Postmarital living arrangements in historically patrilocal settings: integrating household fission and migration perspectives. Demography. 2017;54: 1425–1449. doi: 10.1007/s13524-017-0588-9 28681168 PMC5856169

[58] Diamond-Smith N, Plaza N, Puri M, Dahal M, Weiser SD, Harper CC. Perceived conflicting desires to delay the first birth: A household-level exploration in Nepal. International perspectives on sexual and reproductive health. 2020;46: 125. doi: 10.1363/46e9420 32723708 PMC7433350

[59] Khalil U, Mookerjee S. Patrilocal residence and women’s social status: evidence from South Asia. Economic Development and Cultural Change. 2019;67: 401–438.

[60] Pradhan R, Meinzen-Dick R, Theis S. Property rights, intersectionality, and women’s empowerment in Nepal. Journal of Rural Studies. 2019;70: 26–35.

[61] Furuta M, Salway S. Women’s position within the household as a determinant of maternal health care use in Nepal. International family planning perspectives. 2006; 17–27. doi: 10.1363/3201706 16723298

[62] Diamond-Smith N, Raj A, Prata N, Weiser SD. Associations of women’s position in the household and food insecurity with family planning use in Nepal. PLoS One. 2017;12: e0176127. doi: 10.1371/journal.pone.0176127 28453562 PMC5409177

[63] Morrison J, Giri R, Arjyal A, Kharel C, Harris-Fry H, James P, et al. Addressing anaemia in pregnancy in rural plains Nepal: A qualitative, formative study. Maternal & child nutrition. 2021;17: e13170. 34241951 10.1111/mcn.13170 PMC8269150

[64] Shahabuddin A, Delvaux T, Nöstlinger C, Sarker M, Bardají A, Sharkey A, et al. Maternal health care-seeking behaviour of married adolescent girls: A prospective qualitative study in Banke District, Nepal. PloS one. 2019;14: e0217968. doi: 10.1371/journal.pone.0217968 31237907 PMC6592531

[65] Shri N, Muhammad T. Association of intimate partner violence and other risk factors with HIV infection among married women in India: Evidence from National Family Health Survey 2015–16. BMC public health. 2021;21: 1–11. doi: 10.1186/s12889-020-10013-y 34789185 PMC8597306

[66] Hershow RB, Bhadra M, Mai NVT, Sripaipan T, Ha TV, Go VF. A qualitative study with women living with HIV on perceived gender norms and experiences of intimate partner violence in Northern Vietnam. Journal of interpersonal violence. 2020;35: 5905–5925. doi: 10.1177/0886260517724834 29294869

[67] Baker C, Limato R, Tumbelaka P, Rewari BB, Nasir S, Ahmed R, et al. Antenatal testing for anaemia, HIV and syphilis in Indonesia—A health systems analysis of low coverage. BMC Pregnancy and Childbirth. 2020;20. doi: 10.1186/s12884-020-02993-x 32471383 PMC7257553

[68] Crozier K, Chotiga P, Pfeil M. Factors influencing HIV screening decisions for pregnant migrant women in South East Asia. Midwifery. 2013;29: e57–e63. doi: 10.1016/j.midw.2012.08.013 23245457

[69] Karkee R, Lee AH, Pokharel PK. Women’s perception of quality of maternity services: a longitudinal survey in Nepal. BMC pregnancy and childbirth. 2014;14: 1–7. doi: 10.1186/1471-2393-14-1 24456544 PMC3902186

[70] Mehata S, Paudel YR, Dariang M, Aryal KK, Paudel S, Mehta R, et al. Factors determining satisfaction among facility-based maternity clients in Nepal. BMC pregnancy and childbirth. 2017;17: 1–10. 28946851 10.1186/s12884-017-1532-0 PMC5613378

[71] Awasthi MS, Awasthi KR, Thapa HS, Saud B, Pradhan S, Khatry RA, et al. Utilization of antenatal care services in Dalit communities in Gorkha, Nepal: a cross-sectional study. Journal of pregnancy. 2018;2018. doi: 10.1155/2018/3467308 30515327 PMC6236651

[72] Thapa P, Bangura AH, Nirola I, Citrin D, Belbase B, Bogati B, et al. The power of peers: an effectiveness evaluation of a cluster-controlled trial of group antenatal care in rural Nepal. Reproductive health. 2019;16: 1–14. 31640770 10.1186/s12978-019-0820-8 PMC6805428

